# Systemic Inflammatory Response Syndrome After Extracorporeal Membrane Oxygenation Decannulation in COVID-19 Patients

**DOI:** 10.7759/cureus.36436

**Published:** 2023-03-20

**Authors:** Pradhab Kirupaharan, Cameron Blazoski, Robert Hilton, Eric Feduska, Ron Leong, Michael Baram

**Affiliations:** 1 Pulmonary and Critical Care Medicine, Thomas Jefferson University Hospital, Philadelphia, USA; 2 Internal Medicine, Sidney Kimmel Medical College, Philadelphia, USA; 3 Anesthesiology and Critical Care, Thomas Jefferson University Hospital, Philadelphia, USA

**Keywords:** survival, infection, covid-19, sirs, ecmo

## Abstract

Introduction: Systemic inflammatory response syndrome (SIRS) is frequently observed following decannulation from extracorporeal membrane oxygenation (ECMO). Differentiating cytokine release due to infection from endothelial injury from cannula removal and/or discontinuation from the ECMO circuit has been shown to impact treatment and outcomes. This response, however, may be complicated in COVID-19 patients due to prevalent glucocorticoid and immune modulator use. It remains unclear whether COVID-19 infection and/or associated immune modulator use impact the incidence of SIRS following decannulation.

Objectives: The aim of this study is to investigate the incidence of the SIRS phenomenon and associated outcomes in patients with COVID-19 after ECMO decannulation.

Methods: An IRB-approved retrospective chart review of all patients who survived ECMO between June 31, 2010 and July 7, 2021 was done to identify patients who experienced SIRS within 48 hours of decannulation from ECMO support. Patients with COVID-19 were confirmed by a positive reverse transcription polymerase chain reaction (RT-PCR) assay for SARS-CoV2. SIRS was confirmed when two out of three of the following criteria were met: fever, leukocytosis, and/or initiation/escalation of vasopressors. Patients who developed post-ECMO SIRS were then distinguished based on the presence of infection. Infection was defined by the presence of either a new or positive culture following decannulation. We compared the incidence of SIRS and infection within 48 hours of decannulation in patients with and without COVID-19.

Results: We identified 227 eligible patients who survived ECMO. Twenty-eight patients (12%) had COVID-19. Of these patients, ten patients with COVID-19 (36%) experienced post-ECMO SIRS, including those with true SIRS (n=3) and associated infections (n=7). Five of the ten patients with COVID-19 who experienced post-ECMO SIRS were exposed to immune modulators within two weeks of decannulation. Ninety-five (42%) patients without COVID-19 developed post-ECMO SIRS. Thirty-day survival in COVID patients who experienced post-ECMO SIRS compared to COVID patients who did not experience post-ECMO SIRS was 73% vs. 94%. (p=0.11).

Conclusion: Post-ECMO SIRS is common. The incidence of SIRS following decannulation was similar when historically compared to non-COVID patients who survived ECMO in a previously reported cohort from our institution. Immune-modulation exposure within two weeks of decannulation did not affect the incidence of SIRS in patients with COVID-19.

## Introduction

Extracorporeal membrane oxygenation (ECMO) is a technology most often used to support patients with acute severe cardiac and/or respiratory failure and high mortality risk despite the use of maximal conventional therapy [1-3]. In the midst of a pandemic, ECMO was increasingly being used in patients worldwide with coronavirus disease 2019 (COVID-19) [4-6]. ECMO is intended to augment oxygenation and gas exchange with or without hemodynamic support to allow time for end-organ recovery. Patients are ultimately decannulated from ECMO once criteria for adequate tissue perfusion and organ recovery are met [6]. However, there are numerous complications that may occur after weaning from extracorporeal support, which may hinder subsequent recovery [6]. Development of systemic inflammatory response syndrome (SIRS) while on ECMO can occur due to a complex and multi-faceted innate inflammatory response to the foreign extracorporeal circuit [5-8]. Systemic inflammation can also occur due to the underlying disease process or secondary insults related to infection, prolonged ventilator support, aspiration, or extensive vascular injury [6-8]. SIRS has also been observed and, more recently, described in the literature following ECMO decannulation. Poorer outcomes are associated with those who have evidence of true infection and associated sepsis following decannulation [4]. It remains unclear whether COVID-19 or associated treatment with immune-modulating therapies affect the incidence of post-decannulation SIRS or infection. This retrospective chart review was conducted in order to identify and compare the incidence, risk factors, and associated outcomes of SIRS and infection following decannulation from ECMO in patients with and without COVID-19. This article was previously presented as a meeting abstract at the 2022 American Thoracic Society International Conference on May 15, 2022.

## Materials and methods

We conducted an IRB-approved retrospective chart review of all patients who survived ECMO at Thomas Jefferson University Hospital between June 31, 2010 and July 7, 2021. Patients who died within 24 hours of ECMO cannulation were excluded. Patients who died within 48 hours of decannulation were also excluded since these deaths were presumably attributed to preceding organ failure or complications rather than the SIRS response. Patients with COVID-19 were confirmed by a positive reverse transcription polymerase chain reaction (RT-PCR) assay for SARS-CoV2. Per institutional protocol, all patients were confirmed to be afebrile without evidence of uncontrolled or active infection at the time of ECMO decannulation. ECMO decannulation was initially exclusively performed in the operating room with direct vessel repair; however, during COVID, bedside decannulation was increasingly performed to optimize resource utilization. All patients received perioperative antibiotics post-decannulation for 24 hours unless a predetermined course for controlled infection was being completed. SIRS was confirmed when two out of three of the following criteria (fever [>101.5 ℉], leukocytosis [white blood cell > 12,000 or 25% increase from prior baseline], and/or initiation/escalation of vasopressors) were met within 48 hours of decannulation. Conventional SIRS criteria, including tachycardia and tachypnea, were not used due to potential confounding effects related to concurrent analgesia and sedation, vasopressors, inotropes, neuromuscular blocking agents, deconditioning, or specific ventilator settings. Patients who developed post-ECMO SIRS were then distinguished based on the presence of infection. Infection was defined by either (a) a previously recognized infection during ECMO that was appropriately controlled at the time of decannulation or (b) new positive culture data from blood, sputum, urine, or stool assays following decannulation. Patients who developed SIRS criteria without infection were further characterized as having "true SIRS." Administration of immune modulating therapies, including tocilizumab, baricitinib, hydroxychloroquine, and glucocorticoids, during the treatment course for COVID-19 infection was also noted. Veno-arterial ECMO (VA-ECMO) via femoral cannulation was primarily used for patients with cardiogenic shock, whereas the majority of patients with respiratory failure underwent veno-venous ECMO (VV-ECMO) using either a single-site, dual-lumen right internal jugular (IJ) venous cannulation or traditional dual-site cannulation via the femoral and right IJ venous cannulations. A minority of patients with predominant respiratory failure received VA-ECMO due to concurrent heart failure, hypotension, or anatomical or technical limitations. ECMO circuits included a Rotaflow centrifugal pump (Maquet, Rastatt, Germany) and a Quadrox-D diffusion membrane hollow-fiber oxygenator (Maquet, Rastatt, Germany).

Statistical analysis

Data were expressed as a number paired with the percentage, mean, or median (quantile), as appropriate. The two groups were compared using chi-squared tests for categorical variables and standard t-tests for continuous variables. Statistical significance was accepted at a level of p = 0.05.

## Results

We identified 227 eligible patients who survived ECMO. Twenty-eight patients (12%) had COVID-19. Of these, ten patients with COVID-19 experienced post-ECMO SIRS (36%), with a subset of five patients (50%) exposed to immune-modulating therapies within two weeks of decannulation. Among the ten with COVID-19 and SIRS, three had "true SIRS" (30%) and seven had SIRS associated with infection (70%).

Patients with COVID-19 who experienced post-ECMO SIRS had lower 30-day survival compared to patients with COVID-19 who did not experience post-ECMO SIRS (70% vs. 94%, p=0.09). Patients with COVID-19 who experienced post-ECMO SIRS were significantly older (54.6 vs. 46.2, p = 0.026); however, there were no other significant differences in other baseline demographics, pre-ECMO vital signs, pre-ECMO or pre-decannulation laboratory values, or listed complication rates (Table 1). There was no difference in ECMO duration (20.7 days vs. 19.5 days, p=0.87) when comparing COVID patients who developed SIRS and those who did not. Additionally, immune modulator exposure within two weeks of decannulation did not increase the risk of developing SIRS among patients with COVID-19 on ECMO (OR 0.89, 95% CI 0.186-4.224, p=0.883).

**Table 1:** Comparison of patients with COVID-19 with/without post-decannulation systemic inflammatory response syndrome ECMO: extracorporeal membrane oxygenation, VA: venoarterial; VV: veno-venous; FiO_2_: fraction of inspired oxygen; PEEP: positive end expiratory pressure; SIRS: systemic inflammatory response syndrome

	COVID+ with SIRS (n=10)	COVID+ without SIRS (n=17)	P-values
Pre-ECMO demographics			
Age (years)	56.2 ± 8.0	46.2 ± 11.8	0.026
Male	7 (70%)	12 (71%)	0.974
Body surface area (cm^2^)	2.2 ± 0.2	2.0 ± 0.3	0.073
Body mass index	35.5 ± 8.0	34.0 ± 6.9	0.611
ECMO strategy			
VA	0 (0%)	0 (0%)	1.000
VV	10 (100%)	17 (100%)	1.000
Length on ECMO (days)	20.7 ± 12.3	19.5 ± 20.4	0.868
Comorbidities			
Smoking	1 (10%)	2 (12%)	0.888
Coronary artery disease	0 (0%)	0 (0%)	1.000
Diabetes mellitus	2 (20%)	4 (24%)	0.831
Pre-ECMO vitals signs			
Temperature (°F)	99.9 ± 1.5	99.3 ± 1.6	0.345
Heart Rate	102.1 ± 28.2	107.5 ± 21.0	0.575
Respiratory rate	30.3 ± 5.9	27.4 ± 4.0	0.139
Mean arterial pressure (mmHg)	80.1 ± 17.1	85.6 ± 11.3	0.323
FiO_2_ (%)	93.0 ± 14.9	91.8 ± 12.4	0.823
PEEP (cm)	14.8 ± 3.6	15.1 ± 5.2	0.874
Pre-ECMO laboratory data			
White blood cell count (B/L)	15.7 ± 8.2	15.0 ± 11.5	0.868
Creatinine (mg/dl)	1.0 ± 0.3	1.2 ± 1.5	0.683
Bilirubin (mg/dl)	0.7 ± 0.6	0.8 ± 1.0	0.777
Aspartate aminotransferase (IU/L)	58.2 ± 31.1	62.1 ± 30.0	0.750
Alanine aminotransferase (IU/L)	46.2 ± 30.2	65.3 ± 60.9	0.365
Lactate (mmol/L)	1.5 ± 0.7	1.7 ± 0.7	0.480
Pre-decannulation laboratory data			
White blood cell count (B/L)	14.5 ± 6.4	13.6 ± 5.7	0.708
Creatinine (mg/dl)	1.1 ± 0.6	1.1 ± 1.1	0.999
Bilirubin (mg/dl)	0.6 ± 0.2	0.9 ± 0.9	0.312
Aspartate aminotransferase (IU/L)	60.1 ± 27.8	85.3 ± 118.0	0.516
Alanine aminotransferase (IU/L)	45.2 ± 28.2	80.1 ± 101.3	0.300
Lactate (mmol/L)	1.5 ± 0.3	1.4 ± 0.4	0.501
Complications during ECMO			
Acute kidney injury	2 (20%)	0 (0%)	0.055
Acute liver failure	0 (0%)	0 (0%)	1.000
Stroke	0 (0%)	0 (0%)	1.000
Intracranial hemorrhage	0 (0%)	1 (6%)	0.434
Cannulation site bleed	1 (10%)	1 (6%)	0.693
Gastrointestinal hemorrhage	1 (10%)	3 (18%)	0.589
SIRS phenomenon			
Fever 24 hours post-decannulation	9 (90%)	5 (29%)	0.002
Leukocytosis 24 hours post-decannulation	3 (30%)	0 (0%)	0.016
Vasopressor use	6 (60%)	5 (29%)	0.118
Outcomes			
30-day survival	7 (70%)	16 (94%)	0.088

When further analyzing the subgroup of COVID-19 patients who developed post-ECMO SIRS, there was no significant difference between patients who developed an infection and those who had true SIRS. The number of patients was insufficient to demonstrate a significant difference in 30-day survival when comparing true SIRS to infection-associated SIRS (66% vs. 75%, p=0.782) (Table 2).

**Table 2:** Subgroup analysis of COVID+ Patients who developed systemic inflammatory response syndrome response ECMO: extracorporeal membrane oxygenation, VA: venoarterial; VV: veno-venous; FiO_2_: fraction of inspired oxygen; PEEP: positive end expiratory pressure; SIRS: systemic inflammatory response syndrome

	True SIRS (n=3)	Infection (n=7)	P-value
Pre-ECMO demographics			
Age (years)	55.7 ± 7.2	56.4 ± 8.8	0.907
Male	3 (100%)	4 (57%)	0.175
Body surface area (cm^2^)	2.1 ± 0.1	2.2 ± 0.3	0.599
Body mass index	33.3 ± 4.2	36.5 ± 9.3	0.692
ECMO strategy			
VA	0 (0%)	0 (0%)	1.000
VV	3 (100%)	7 (100%)	1.000
Length of ECMO (days)	13.7 ± 6.0	23.7 ± 13.5	0.264
Comorbidities			
Smoking history	1 (33%)	0 (0%)	0.107
Coronary artery disease	0 (0%)	0 (0%)	1.000
Diabetes mellitus	1 (33%)	1 (14%)	0.490
Pre-ECMO vital signs			
Temperature (°F)	99.9 ± 1.7	99.9 ± 1.6	1.000
Heart rate	98.3 ± 44.8	103.7 ± 22.7	0.799
Respiratory rate	30.3 ± 4.5	30.3 ± 6.8	1.000
Mean arterial pressure (mmHg)	81.7 ± 8.1	79.4 ± 20.4	0.859
FiO_2_ (%)	90.0 ± 17.3	94.3 ± 15.1	0.701
PEEP (cm)	14.0 ± 5.3	15.1 ± 3.0	0.679
Pre-ECMO laboratory data			
White blood cell count (B/L)	16.8 ± 10.7	15.2 ± 7.9	0.796
Creatinine (mg/dl)	0.9 ± 0.3	1.0 ± 0.3	0.642
Bilirubin (mg/dl)	0.5 ± 0.2	0.8 ± 0.7	0.499
Aspartate aminotransferase (IU/L)	47.3 ± 32.7	62.9 ± 31.8	0.500
Alanine aminotransferase (IU/L)	50.3 ± 47.0	44.4 ± 24.8	0.795
Lactate (mmol/L)	1.1 ± 0.5	1.7 ± 0.8	0.271
Pre-decannulation laboratory data			
White blood cell count (B/L)	14.7 ± 10.9	14.5 ± 4.6	0.967
Creatinine (mg/dl)	0.9 ± 0.4	1.1 ± 0.6	0.617
Bilirubin (mg/dl)	0.4 ± 0.1	0.7 ± 0.1	0.003
Aspartate aminotransferase (IU/L)	42.7 ± 25.1	67.6 ± 27.0	0.211
Alanine aminotransferase (IU/L)	39.7 ± 33.1	47.6 ± 28.8	0.712
Lactate (mmol/L)	1.3 ± 0.4	1.6 ± 0.3	0.222
Complications during ECMO			
Acute kidney injury	0 (0%)	2 (29%)	0.301
Acute liver failure	0 (0%)	0 (0%)	1.000
Stroke	0 (0%)	0 (0%)	1.000
Intracranial hemorrhage	0 (0%)	0 (0%)	1.000
Cannula site bleed	1 (33%)	0 (0%)	0.107
GI hemorrhage	0 (0%)	1 (14%)	0.490
SIRS phenomenon			
Fever 24 hours post-decannulation	3 (100%)	6 (86%)	0.490
Leukocytosis 24 hours post-decannulation	1 (33%)	2 (29%)	0.880
New infection	0 (0%)	4 (57%)	0.091
Vasopressor use	1 (33%)	5 (71%)	0.259
Outcomes			
30-day survival	2 (66%)	5 (71%)	0.880

Ninety-five out of 227 patients without COVID-19 developed post-decannulation SIRS. The risk of developing SIRS was not statistically different compared to those with COVID-19 infection (OR 0.70, 95% CI 0.31-1.58; p=0.385). Patients with COVID-19 who developed SIRS tended to be older (56.2 vs. 49.6 years, p=0.039) and on ECMO longer (20.7 vs. 10.7 days, p=0.031) compared to those with SIRS but without COVID-19. Despite this, there was no statistically significant difference in 30-day mortality between these groups (70% vs. 88%, p=0.131). Additionally, there were no other significant differences in other baseline demographics, pre-ECMO vital signs, comorbidities, or listed complications (Table 3).

**Table 3:** Comparison of patients who experienced systemic inflammatory response syndrome in those with/without preceding COVID-19 infection ECMO: extracorporeal membrane oxygenation, VA: venoarterial; VV: veno-venous; FiO_2_: fraction of inspired oxygen; PEEP: positive end expiratory pressure; SIRS: systemic inflammatory response syndrome

	COVID positive SIRS (n=10)	COVID negative SIRS (n=96)	P-values
Pre-ECMO demographics			
Age (years)	56.2 ± 8.0	49.6 ± 14.3	0.039
Male	7 (70%)	67 (70%)	0.989
Body surface area (cm^2^)	2.2 ± 0.2	2.1 ± 0.3	0.999
Body mass index	35.5 ± 8.0	32.2 ± 8.9	0.245
ECMO strategy			
VA	0 (0%)	63 (66%)	<0.001
VV	10 (100%)	23 (34%)	<0.001
Length on ECMO (days)	20.7 ± 12.3	10.7 ± 5.5	0.031
Comorbidities			
Smoking history	1 (10%)	33 (34%)	0.116
Coronary artery disease	0 (0%)	36 (38%)	0.017
Diabetes mellitus	2 (20%)	22 (23%)	0.834
Pre-ECMO vital signs			
Temperature (°F)	99.9 ± 1.5	97.9 ± 4.9	0.006
Heart rate	102.1 ± 28.2	99.0 ± 36.5	0.754
Respiratory rate	30.3 ± 5.9	22.0 ± 8.4	0.002
Mean arterial pressure (mm Hg)	80.1 ± 17.1	70.9 ± 24.4	0.146
FiO_2_ (%)	93.0 ± 14.9	93.7 ± 16.7	0.891
PEEP (cm)	14.8 ± 3.6	11.5 ± 6.4	0.024
Pre-ECMO laboratory data			
White blood cell count (B/L)	15.7 ± 8.2	14.6 ± 7.8	0.694
Creatinine (mg/dl)	1.0 ± 0.3	1.6 ± 0.9	<0.001
Bilirubin (mg/dl)	0.7 ± 0.6	1.2 ± 1.6	0.057
Aspartate aminotransferase (IU/L)	58.2 ± 31.1	358.7 ± 1051.1	0.006
Alanine aminotransferase (IU/L)	46.2 ± 30.2	277.9 ± 1003.6	0.027
Lactate (mmol/L)	1.5 ± 0.7	5.1 ± 4.8	<0.001
Pre-decannulation laboratory data			
White blood cell count (B/L)	14.5 ± 6.4	15.3 ± 6.4	0.715
Creatinine (mg/dl)	1.1 ± 0.6	1.4 ± 1.6	0.242
Bilirubin (mg/dl)	0.6 ± 0.2	2.0 ± 2.8	<0.001
Aspartate aminotransferase (IU/L)	60.1 ± 27.8	67.2 ± 46.7	0.489
Alanine aminotransferase (IU/L)	45.2 ± 28.2	64.4 ± 59.8	0.092
SIRS phenomenon			
Fever 24 hours post-decannulation	9 (90%)	87 (91%)	0.949
Leukocytosis 24 hours post-decannulation	3 (30%)	72 (75%)	0.003
New infection	4 (40%)	37 (39%)	0.928
Outcomes			
30-day survival	7 (70%)	84 (88%)	0.131

## Discussion

This is the first study to further describe the incidence and clinical implications of SIRS following decannulation from ECMO in patients with COVID-19. The clinical correlation between ECMO and systemic inflammation has been well described in the literature [8]. The introduction of blood into an extracorporeal system is known to incite a robust innate humoral and cellular cascade that can closely resemble SIRS. Leukocyte function, complement activation, and endothelial and thrombotic dysregulation are among the many established downstream effects [6-10]. This interplay between coagulation, complement, and endothelial systems initiates and perpetuates a pro-inflammatory cellular and cytokine milieu, which, if unchecked, can lead to vascular and end-organ injury [10-16].

This study demonstrated that SIRS was commonly observed following decannulation from ECMO support, with an overall incidence of approximately 44% in this cohort. The incidence was similar when comparing those with and without concomitant COVID-19 infection. Despite COVID-19 patients being older and enduring longer durations of ECMO support, neither of these variables significantly impacted the incidence of SIRS response or relevant outcomes. It was initially hypothesized that prolonged cannulation would result in endothelial activation, which might sustain pro-inflammatory activation. It was additionally considered that the severe inflammatory state associated with COVID-19 might predispose to more frequent SIRS following decannulation [8]. These data do not confirm either of these hypotheses. It is unclear whether these results could have been mitigated by other variables.

Among those who developed SIRS, patients with COVID-19 tended to have lower creatinine, hepatic aminotransferase, and lactate levels prior to ECMO cannulation. However, there was no sustained difference in these markers prior to decannulation, which may have contributed to comparable rates of SIRS and subsequent 30-day mortality.

When evaluating patients with COVID-19 more closely, we did not identify any pertinent pre-ECMO or pre-decannulation variable, other than age, which was associated with an increased risk of developing post-ECMO SIRS. Advanced age, in contrast to relevant studies in cardiac surgery, was associated with an increased risk of an inflammatory response and resultant SIRS. Despite an older cohort, SIRS was not associated with worse outcomes, which is consistent with a previous study of non-COVID patients at our institution [1]. There was similar use of adjunctive therapies (i.e., tocilizumab, baricitinib, hydroxychloroquine, and glucocorticoids) within two weeks of decannulation in all patients with COVID-19, suggesting no demonstrable impact of these therapies on SIRS in this cohort.

When evaluating the SIRS response further, the majority of patients with COVID-19 developed SIRS in the context of an associated infection, whereas only three patients developed "true SIRS." Unlike our previous experience with post-ECMO SIRS in patients without COVID-19, the development of post-ECMO SIRS with infection was not associated with worse 30-day survival.

Limitations

This study has several limitations owing to its retrospective methodology, small sample size, and single-institution experience. Furthermore, our definition of SIRS included the initiation/escalation of vasopressors, which varies from conventional definitions and excludes patients with associated tachycardia or tachypnea.

## Conclusions

Post-ECMO SIRS is common. The incidence of SIRS, whether infectious or not, is similar in COVID patients and non-COVID patients who survive ECMO. Immune-modulation exposure within two weeks of decannulation did not affect the incidence of SIRS in patients with COVID-19.
